# Fim3-dependent autoagglutination of *Bordetella pertussis*

**DOI:** 10.1038/s41598-023-34672-0

**Published:** 2023-05-10

**Authors:** Nao Otsuka, Kentaro Koide, Masataka Goto, Kazunari Kamachi, Tsuyoshi Kenri

**Affiliations:** Department of Bacteriology II, National Institute of Infectious Diseases, 4-7-1 Gakuen, Musashimurayama, Tokyo 208-0011 Japan

**Keywords:** Bacteria, Bacterial genetics, Cellular microbiology

## Abstract

Autoagglutination (Agg) of *Bordetella pertussis* is often observed in clinical laboratory. However, its causal factors and frequency in circulating strains are unknown. Repeated single colony isolation enabled us to detect an Agg^-^ mutant in the supernatant of an Agg^+^ strain of *B. pertussis*. Whole-genome sequencing and immunoblot analysis disclosed that the Agg^-^ mutant had a single C-deletion in its *fim3* promoter region (Pfim3) which abolished Fim3 fimbriae production. A *B. pertussis fim3*-knock out mutant also lacked the Agg^+^ phenotype. Agg^+^ clinical isolates were detected a higher production of Fim3 than Fim3-producing Agg^-^ isolates. *B. pertussis* is known to harbor multiple Pfim3 poly(C) lengths within a single strain culture and our newly developed PCR/LDR assay revealed that Agg^+^ isolates harbor the highest Pfim3 poly-14C abundance. We evaluated the frequency of autoagglutination in clinical *B. pertussis* isolates collected in Japan between 1994 and 2018 (n = 203). Fim3 production was confirmed for 190 isolates and 74.7% of them displayed the Agg^+^ phenotype. The Agg^+^ phenotype was strongly associated with Pfim3 poly-14C abundance. Taken together, our findings demonstrated that *B. pertussis* autoagglutination occurs in response to high Fim3 levels and the Agg^+^ strain has predominated in Japan over the past two decades.

## Introduction

Bacterial autoagglutination (Agg) is a widespread phenomenon caused by several environmental and pathogenic bacteria such as *Pseudomonas putida*, *Escherichia coli*, and *Legionella pneumophila*. Nevertheless, little is known about its mechanisms and causal factors^1,2^. Autoagglutination of *Bordetella pertussis*, the human respiratory pathogen, is familiar to clinicians but the factors causing it and its frequency of occurrence in circulating *B. pertussis* strains are unknown. Previously, it was believed that hydrophobic interactions between cell surface proteins (filamentous hemagglutinin, FHA) responsible for this phenomenon as these interactions were abolished by adding cyclodextrin to liquid synthetic medium^3^. However, cyclodextrin did not inhibit autoagglutination when we examined clinical *B. pertussis* isolates in Japan. Moreover, even an FHA-deficient *B. pertussis* isolate underwent autoagglutination. Hence, a crucial factor other than FHA is implicated in *B. pertussis* autoagglutination. Furthermore, *B. pertussis* autoagglutination did not occur in the presence of modulating substances such as MgSO_4_ and nicotinic acid. It suggested that the unknown autoagglutination factor is under the regulation of BvgAS two-component system.

Most of virulence factors of *B. pertussis* including the fimbriae (Fim) are controlled by the BvgAS two-component system comprising the BvgS sensor kinase and the BvgA response regulator. *B. pertussis* produces either one or both serologically distinct fimbriae designated Fim2 and Fim3 (22.5 kDa and 22.0 kDa, respectively) and their production patterns have been used in strain serotyping^4,5^. *B. pertussis* isolates can be typed as 4 variants include Fim2, Fim2/3, Fim3, and Fim^-^. The genes *fim2* and *fim3* encode the major fimbrial subunits and are localized to different transcriptional units separated by a distance of 471 kb, and their expression requires binding of phosphorylated BvgA dimers to sites overlapping the gene promoter region^6^. In addition to the regulation by BvgAS, *fim2* and *fim3* also independently undergo phase variation through alteration of the lengths of the monotonic cytosine residue stretches in their promoter regions (Pfim poly(C)). When poly(C) is too long or too short, the RNA polymerase binding sites will be unable to bind the promoter-proximal BvgA ~ P and fimbrial transcription will not be initiated. This defect occurs when a single C-residue is inserted or deleted^6,7^.

The present study explored the crucial factor regulating *B. pertussis* autoagglutination. We compared whole-genome sequences of Agg^+^ isolate and its cognate Agg^-^ mutant. A single C-deletion occurred in the promoter region of *fim3* (Pfim3) of Agg^-^ mutant and abolished Fim3 production. A genetically engineered *B. pertussis fim3*-knockout mutant also lacked Agg^+^ phenotype, indicating *B. pertussis* autoagglutination is Fim3-dependent. We previously showed that Fim3 protein is produced in clinical *B. pertussis* isolates with Pfim3 poly(C) ≥ 14C^8^. This finding and the discoveries made herein clarified the relationship between autoagglutination and Pfim3 poly(C) length. We used a newly developed PCR/LDR assay to investigate the distribution and abundance ratios of each Pfim3 poly(C) length in clinical *B. pertussis* isolates. We also evaluated the Pfim3 poly(C) distribution and the prevalence of the Agg^+^ strain in 203 clinical *B. pertussis* isolates collected in Japan between 1994 and 2018.

## Results

### Autoagglutination of B. pertussis isolates

**Figure 1:**
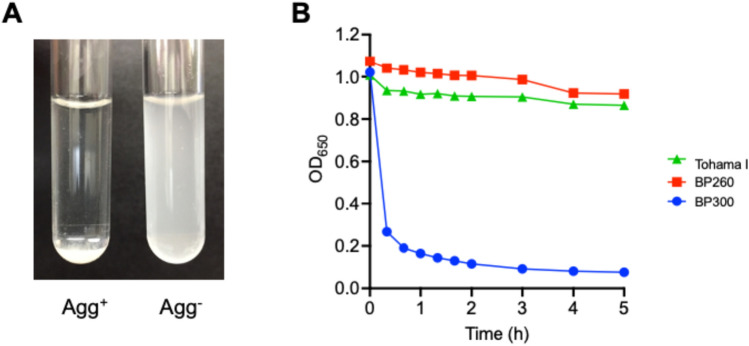
In vitro autoagglutination of *Bordetella pertussis*. *B. pertussis* isolates were suspended in 1% (w/v) casamino acid solution and their OD650 was monitored. (**A**) After 5-h static incubation, the Agg^+^ strain sedimented (left) whereas the Agg^-^ strain retained its initial turbidity (right). (**B**) *B. pertussis* Agg^+^ isolate (BP300) and Agg^-^ isolates (Tohama I and BP260) were cultured on CSM plates and bacterial suspension turbidity was measured in terms of OD650 value.

When the bacterial suspension was statically incubated, the Agg^+^ strain formed aggregates and sedimented at the bottom of the test tube whereas the Agg^-^ strain did not (Fig. 1A). *B. pertussis* isolates were suspended in 1% (w/v) casamino acid solution and their OD_650_ were measured for 5 h. Here, autoagglutination was defined as ≥ 40% reduction in the OD_650_ of the bacterial suspension after 5 h static incubation. Figure 1B shows representative changes in the turbidity of *B. pertussis* Agg^+^ or Agg^-^ suspensions. The Agg^+^ isolate BP300 rapidly sedimented within the first 20 min and its OD_650_ declined by 92.6% after 5 h of static incubation. However, the Agg^-^ isolates Tohama I and BP260 did not autoagglutinate and they retained their initial OD_650_ values.

### Isolation of B. pertussis Agg^-^ mutant from Agg^+^ strain

Agg^+^ strain and its cognate Agg^-^ mutant were required to identify the factor responsible for autoagglutination. We previously hypothesized that the supernatant of the sedimented *B. pertussis* Agg^+^ strain contained planktonic cells that were unable to autoagglutinate.

**Figure 2:**
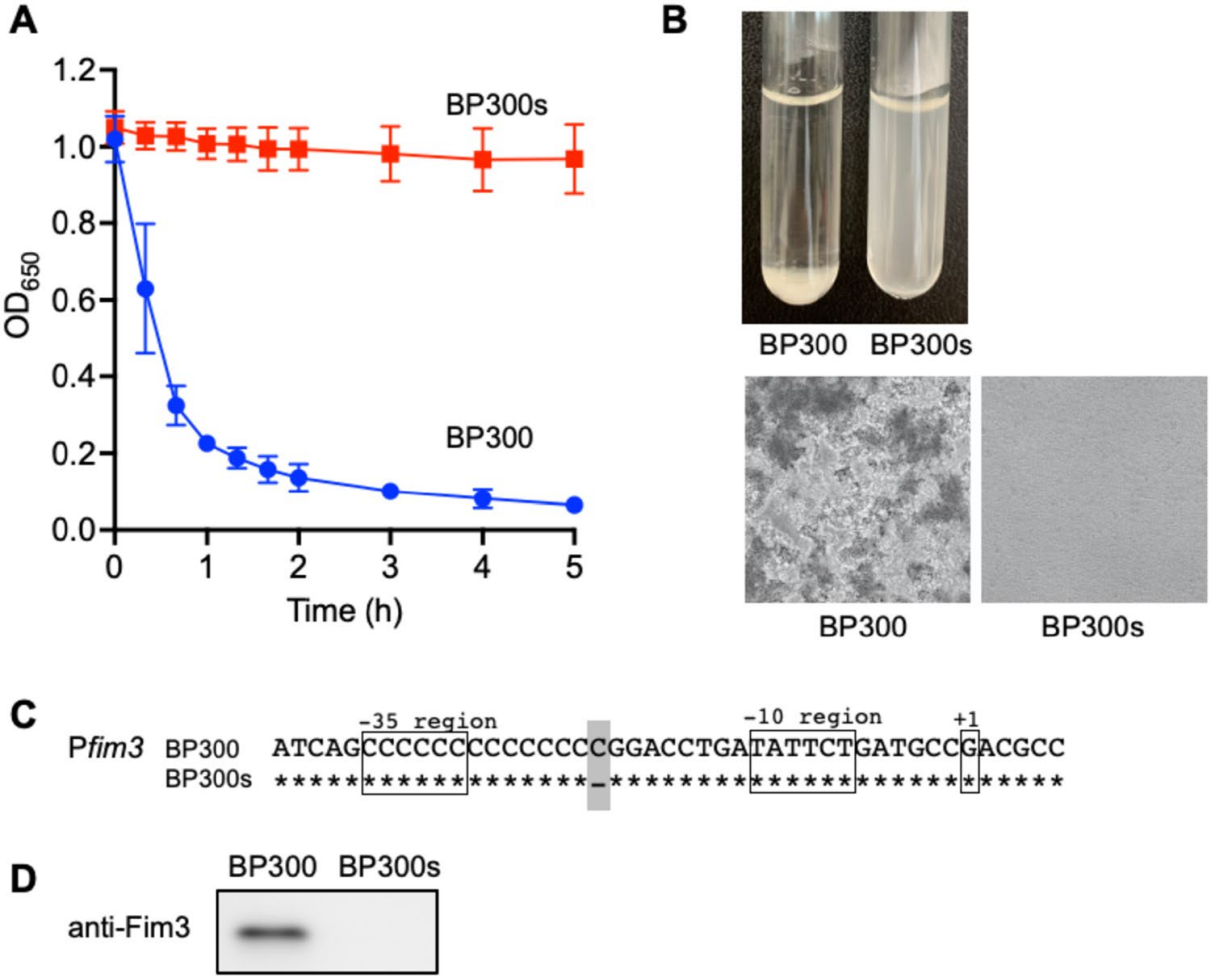
Isolation of the *B. pertussi*s Agg^-^ mutant from the Agg^+^ strain. (**A**) Autoagglutination assay of BP300 and BP300s cultured on CSM plates. (**B**) Autoagglutination was visualized in test tubes after 5 h of static incubation (upper panel). Phase-contrast micrographs after 5 h of static incubation (lower panel). Magnification×100. (**C**) Single-point mutation detected in the fim3 promoter region of Agg^-^ mutant BP300s. The upstream sequences of fim3 are shown and the SNP position is highlighted in gray. The -35, -10, and + 1 positions of the Pfim3 region are indicated based on a previous publication^7^. (**D**) Immunoblot analysis using anti-Fim3 antibody.

By repeating a single-colony isolation twice, we obtained the Agg^-^ mutant from the supernatant of the *B. pertussis* Agg^+^ isolate BP300. The Agg^-^ mutant BP300s did not autoagglutinate within 5 h of static incubation (Fig. 2A). BP300 caused autoagglutination and sedimented at the bottom of the culture tube whereas BP300s remained turbid. BP300 formed large bacterial clumps while BP300s did not (Fig. 2B). *B. pertussis* BP300 and BP300s were subjected to the whole-genome sequencing (WGS) to identify the factor causing autoagglutination in *B. pertussis*. Two of point mutations were detected in the upstream regions of *fim3* and *rpoA* genes in the BP300s genome (Fig. 2C, Supplementary Fig. S1A). The distance between the -10 and the -35 promoter elements determines the strength of *fim3* promoter, and at least 14 bp is required for transcription. BP300 harbored 14C residues in Pfim3 poly(C) tract while BP300s harbored only 13C residues over a non-expressing length. Immunoblot analysis showed that BP300s did not produce Fim3 (Fig. 2D). The mutation in the upstream region of *rpoA* gene did not affect protein production (Supplementary Fig. S1B). The preceding results suggest that there is an association between Fim3 and autoagglutination.

### B. pertussis autoagglutination is Fim3-dependent

**Figure 3:**
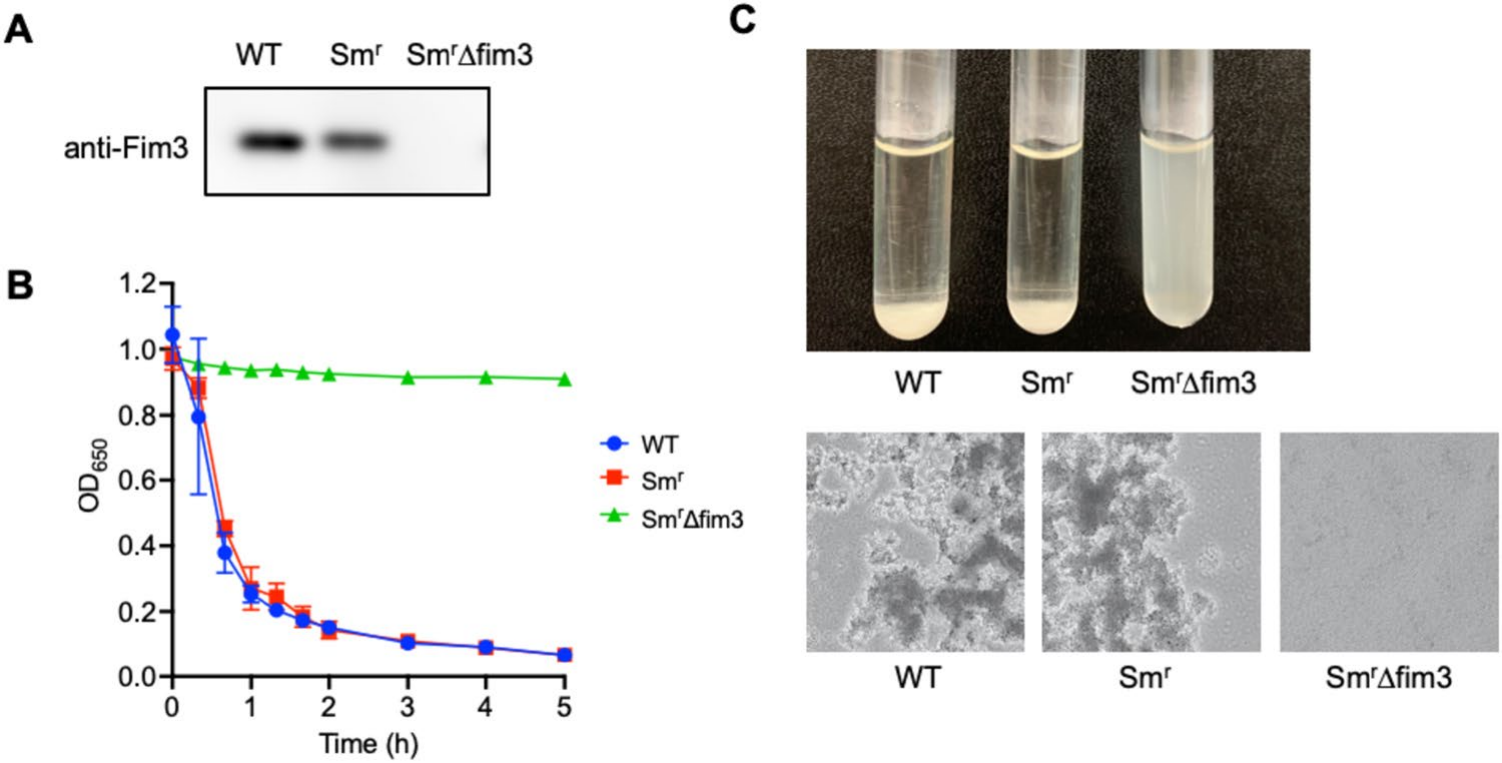
*B. pertussis* autoagglutination is Fim3-dependent. (**A**) Immunoblot analysis using anti-Fim3 antibody. (**B**) Autoagglutination of *B. pertussis* BP300 (WT), BP300Sm^r^ (Sm^r)^, and BP300Sm^r^∆fim3 mutant (Sm^r^∆fim3). (**C**) Bacterial suspensions after 5 h of static incubation (upper panel). Phase-contrast micrographs after 5 h of static incubation (lower panel). Magnification×100.

To determine whether Fim3 is required for *B. pertussis* autoagglutination, we constructed a *B. pertussis* BP300 *fim3*-knockout mutant (Fig. 3A). An autoagglutination assay revealed that ∆fim3 mutant maintained its initial turbidity after 5 h of static incubation and the Agg^+^ phenotype was diminished (Fig. 3B). Figure 3C shows that WT (BP300) and Sm^r^ (streptomycin-resistant BP300) sedimented at the bottom of the culture tube. Phase-contrast images disclosed that large bacterial clumps formed. By contrast, the ∆fim3 mutant did not form bacterial aggregates. Thus, *B. pertussis* autoagglutination depends upon Fim3. Meanwhile, we also investigated the contribution of Fim2 to *B. pertussis* autoagglutination, using *fim2* gene-complemented mutants (Supplementary Fig. S2). The Sm^r^∆fim3_fim2 comp mutant (Fim2^+^/Fim3^-^) did not show autoagglutination, and the Sm^r^_fim2 comp mutant (Fim2^+^/Fim3^+^) induced weaker autoagglutination compared with Sm^r^ (Fim2^-^/Fim3^+^) strain. Therefore, these results suggest that Fim2 does not contribute to *B. pertussis* autoagglutination but likely interfere with it.

### Abundance of Pfim3 poly-14C determines Fim3 production and autoagglutination

**Figure 4:**
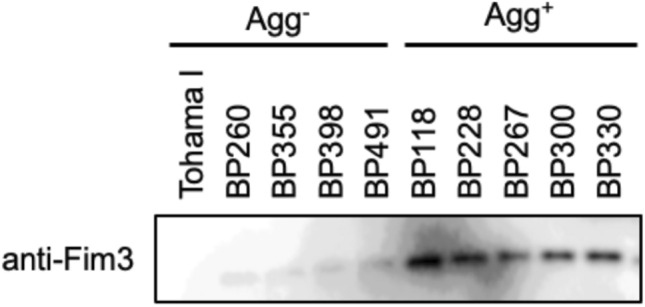
Fim3 production in selected *B. pertussis* isolates. Fim3 production in Agg^-^ strains (Tohama I, BP260, BP355, BP398, and BP491) and Agg^+^ strains (BP118, BP228, BP267, BP300, and BP330) was evaluated by immunoblot analysis.

It was demonstrated that Fim3 is required for *B. pertussis* autoagglutination. Nevertheless, we detected the strains which do not exhibit the Agg^+^ phenotype in Fim3-producing isolates (e.g., BP260 in Fig. 1). We measured Fim3 production in selected isolates to identify the difference between the Agg^+^ and Agg^-^ strains among Fim3-producing isolates (Fig. 4). *B. pertussis* Tohama I is an Agg^-^ strain that does not produce Fim3. The Agg^-^ strains BP260, BP355, BP398, and BP491 and the Agg^+^ strains BP118, BP228, BP267, BP300, and BP330 all produce Fim3. Immunoblot analysis revealed higher Fim3 production in the Agg^+^ strains than the Agg^-^ strains, indicating high Fim3 levels are essential for autoagglutination.

Since *B. pertussis* strain includes multiple different Pfim3 poly(C) lengths in a single cell culture^9^, sequenced chromatograms of Pfim3 region are often ambiguous. To identify the associations among Pfim3 poly(C) length distribution, Fim3 production quantity, and Agg^+^ phenotype, we determined the abundance ratios of each poly(C) length in a mixed sample. We combined the newly developed PCR/LDR assay with GeneScan to estimate the Pfim3 poly(C) length distributions in each *B. pertussis* isolate. The PCR/LDR resolved Pfim3 poly(C) lengths in the range of 11C–16C. The GeneScan produced peak areas corresponding to each poly(C) length and generated abundance ratios for each isolate. The abundance ratios of the Pfim3 poly(C) lengths in selected *B. pertussis* isolates are listed in Table 1. *B. pertussis* Tohama I harbors Pfim3 poly-11C to poly-16C at 9.0%, 34.3%, 43.4%, 12.4%, 3.4%, and 0.4% abundance, respectively. The highest abundance was identified for the poly-13C length in this strain. Fim3-low producing Agg^-^ isolates (i.e., BP260, BP355, BP398, BP491) were detected the highest Pfim3 poly(C) abundance at poly-15C or 16C length. Meanwhile, Fim3-high producing Agg^+^ isolates (i.e., BP118, BP228, BP267, BP300, BP330) were commonly detected the highest abundance at poly-14C length. The poly-14C lengths of the Agg^+^ strains had significantly higher Pfim3 abundance than those of the Agg^-^ strains (*P* < 0.01; Mann–Whitney test).

**Table 1:** Abundance ratio of each Pfim3 poly(C) length in selected *B. pertussis* isolates.

	Fragment size (nt) (Measured size ± SD)^b^	Abundance ratio of each poly(C) length (%; mean ± SD)^a^					
		11C	12C	13C	14C	15C	16C
		40 (32.5 ± 0.20)	43 (35.5 ± 0.06)	46 (38.4 ± 0.12)	49 (41.2 ± 0.12)	52 (44.2 ± 0.10)	55 (47.1 ± 0.08)
Agg^-^ strain	Tohama I	9.0 ± 0.59	34.3 ± 1.25	43.4 ± 4.05	12.4 ± 0.27	3.4 ± 0.39	0.4 ± 0.31
	BP260	0.7 ± 0.57	3.3 ± 1.14	10.4 ± 2.76	26.8 ± 0.90	37.3 ± 5.79	21.6 ± 1.30
	BP355	0.8 ± 0.72	3.4 ± 0.59	10.7 ± 1.16	27.4 ± 1.08	35.9 ± 2.41	22.0 ± 1.31
	BP398	0.0 ± 0.07	0.6 ± 0.04	2.2 ± 0.11	12.0 ± 0.35	36.9 ± 0.43	48.4 ± 0.77
	BP491	0.0 ± 0.08	0.5 ± 0.05	3.0 ± 0.17	14.5 ± 0.75	37.5 ± 0.37	44.5 ± 1.41
Agg^+^ strain	BP118	2.0 ± 0.56	10.6 ± 0.76	25.4 ± 1.10	39.3 ± 1.45	17.6 ± 0.53	5.1 ± 0.48
	BP228	2.5 ± 0.58	11.5 ± 0.61	26.3 ± 1.11	37.7 ± 1.55	17.0 ± 0.53	5.1 ± 0.23
	BP267	2.3 ± 0.25	11.7 ± 0.59	26.4 ± 0.21	37.4 ± 0.43	16.8 ± 0.23	5.3 ± 0.29
	BP300	2.1 ± 0.28	10.9 ± 0.44	26.3 ± 0.72	38.3 ± 0.44	17.3 ± 0.51	5.1 ± 0.31
	BP330	1.9± 0.58	11.0 ± 0.57	25.3 ± 1.08	37.3 ± 0.77	18.6± 1.39	5.9 ± 0.87

^a^Mean abundance ratios for three independent experiments.

^b^Measured fragment sizes are means ± SD for 30 runs.

These results indicate that the abundance of Pfim3 poly-14C regulates the quantity of Fim3 production and subsequent autoagglutination in *B. pertussis*.

### Prevalence of Agg^+^ among B. pertussis strains circulating in Japan

**Figure 5:**
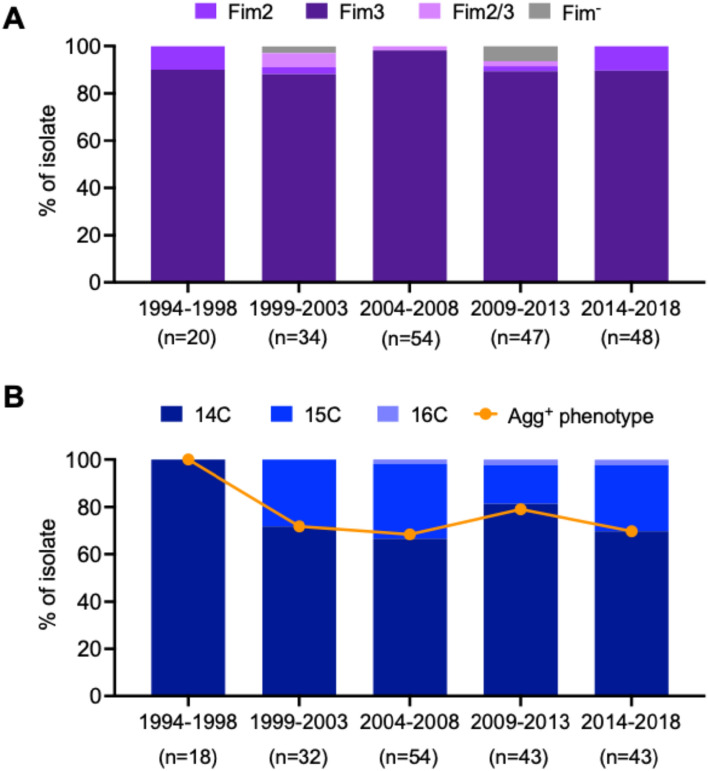
Prevalence of Agg^+^ among the *B. pertussis* strains circulating in Japan. The study was divided into the time intervals 1994–1998, 1999–2003, 2004–2008, and 2014–2018. (**A**) Temporal trend of fimbrial serotypes among clinical *B. pertussis* isolates (n = 203). Serotypes were determined based on Fim2 and Fim3 production patterns and classified into Fim2, Fim3, Fim2/3, and Fim^-^ variants. (**B**) Temporal trend of Agg^+^ strain among Fim2/3 and Fim3 isolates (n = 190). Pfim3 poly(C) distribution in each isolate was analyzed by PCR/LDR assay. Bar graph showing poly(C) length with highest abundance ratio in each isolate. Line graph representing % of Agg^+^ phenotype in each study period.

The prevalence of *B. pertussis* Agg^+^ strain was determined for clinical isolates collected in Japan between 1994 and 2018 (n = 203). The most frequent serotype was Fim3 (91.6%) followed by Fim2, Fim2/3, and Fim^–^ serotypes (4.4%, 2.0%, and 2.0%, respectively) (Fig. 5A). The temporal trend of Agg^+^ phenotype among Fim3-producing isolates (Fim2/3 and Fim3 serotypes; n = 190) was analyzed (Fig. 5B). In the period of 1994–1998 (n = 18), 1999–2003 (n = 32), 2004–2008 (n = 54), 2009–2013 (n = 43), and 2014–2018 (n = 43), the Agg^+^ phenotype was detected in 100.0%, 71.9%, 68.5%, 79.1%, and 69.8% of the isolates, respectively. The frequency of the Agg^+^ phenotype was slightly higher in 1994–1998 than the other 4 periods, but the difference was not statistically significant (*P* > 0.05; one-way ANOVA). Overall, the Agg^+^ phenotype was detected in 74.7% of the Fim3-producing isolates. Hence, the Agg^+^ strain has predominated among *B. pertussis* strains circulating in Japan over the past two decades.

The Pfim3 poly(C) length distributions for each isolate were also determined using PCR/LDR assay. Isolates with the highest Pfim3 poly-14C abundance harbored it at 100%, 71.9%, 66.7%, 81.4%, and 69.8% in each period, respectively. Five isolates from 2004 to 2008 (BP298, 331, 338, 339, and 340; Supplementary Data Set Table S3) demonstrated poor reproducibility in the autoagglutination assay but harbored the highest abundances of Pfim3 poly-15C or poly-16C. The isolates harboring the highest abundance ratios of Pfim3 poly-14C were probably the Agg^+^ phenotype (Fig. 5B). Based on the results obtained for 203 isolates, the lowest Pfim3 poly-14C abundance ratio was 32.3% for the reproducible Agg^+^ phenotype (BP122; Supplementary Data Set Table S3). However, *B. pertussis* BP235 in 2004 harbored the highest abundances of Pfim3 poly-15C (34.8%) and poly-14C (27.7%) but showed the reproducible Agg^+^ phenotype. The Fim3 strain BP416 in 2012 harbored the highest abundance of Pfim3 poly-14C (38.2%) but demonstrated no autoagglutination. The reasons for these exceptions have not yet been established.

## Discussion

The present study showed that Fim3-dependent *B. pertussis* autoagglutination occurs when cells produce high levels of Fim3 protein. The Pfim3 region contains a unique poly(C) sequence and mononucleotide length of the repeat influences *fim3* expression. Our newly developed PCR/LDR assay revealed that a single-strain culture includes multiple Pfim3 poly(C) sequence lengths and that Fim3 production is enhanced when the poly-14C length has the highest abundance ratio in that isolate. Previously, we showed that *B. pertussis* Fim3 strain predominates in Japan^10^ and continuous prevalence of the Fim3 strain was confirmed in the present study. We investigated the serotypes of the *B. pertussis* strains collected between 1994 and 2018 (n = 203) and 93.6% of them were Fim3-producing isolates (Fim3 or Fim2/3 strain). The Agg^+^ phenotype was detected throughout the study period. Overall, 74.7% of the Fim3-producing isolates exhibited the Agg^+^ phenotype. Furthermore, Agg^+^ phenotype strongly associated with the Pfim3 poly-14C abundance ratio of the isolate. *B. pertussis* Fim3 strain has the Agg^+^ phenotype and predominated in Japan over the past two decades, suggesting a selective advantage in environmental fitness for this strain.

*B. pertussis* Fim3 is in the type 1 pilus family. Type 1 fimbriae are long, thin, hair-like structures composed of thousands of pilin subunits^11^. Irons et al. (1985) reported that purified Fim3 is highly hydrophobic and aggregative^12^. Thus, the bacterial autoagglutination observed here might have been induced by direct Fim3-Fim3 interactions. Several prior experiments demonstrated that type 1 fimbriae were implicated in autoagglutination and binding. For instance, expressions of the type 1 fimbriae, Curli and FimH, lead to autoagglutination in *Escherichia coli*^13–15^. Farfan et al. (2011) found that Lpf fimbriae binds the extracellular matrix (ECM) proteins of the host cell. This mechanism contributes to enterohemorrhagic *E. coli* (EHEC) colonization in the gastrointestinal tract^16^. *B. pertussis* aggregates appeared as hydrophobic clots under a light microscope. SEM images showed that Agg^+^ isolates surrounded by the ECM they produced whereas Agg^-^ isolates lacked this envelope (Supplementary Fig. S3). These findings suggest that Fim3 participates in ECM development. *B. pertussis* ECM is a complex structure consisting of polysaccharides such as Bps, extracellular DNA, lipopolysaccharide (LPS), and proteins^17^. It is unknown whether Fim3 directly binds or indirectly stimulates the biosynthesis of these substances. Nevertheless, enhanced ECM development may promote rapid autoagglutination by increasing bacterial aggregate size and weight.

*B. pertussis* Agg^+^ strain has widely circulated in Japan. Therefore, the associations between autoagglutination and bacterial pathogenicity are of great epidemiological concern. The formation of large bacterial aggregates of Agg^+^ strains could be highly significant in this regard. A recent study reported a negative correlation between bacterial aggregate size and phagocytotic efficiency in polynuclear leukocytes (PMNs)^18^. PMNs are components of the innate immune system and the first line of host defense against various microbial pathogens. *Pseudomonas aeruginosa, Staphylococcus aureus, Staphylococcus epidermidis,* and *E. coli* aggregates approximately ≤ 5 µm in diameter were consistently phagocytosed by PMNs. The effect of bacterial aggregate size on phagocytosis, however, might vary with species^19,20^. Future research should investigate this relationship for *B. pertussis*.

We assessed the relevance of Pfim3 poly-14C sequence in autoagglutination using the newly developed PCR/LDR assay. It is often difficult to measure high numbers of mononucleotide repeats because of slippage. Bacterial phase variation sensitively reflects single-nucleotide differences in repeat sequences. Currently, mononucleotide repeats in bacterial genomes are analyzed by (i) Sanger DNA sequencing, (ii) whole-genome sequencing (WGS) and (iii) GeneScan fragment analysis^9,21,22^. We found that Fim3-producing isolates have wide ranges of Pfim3 poly(C) lengths even within a single strain. Therefore, we augmented the discrimination power by combining PCR/LDR with GeneScan. Table 1 shows that the mean inter-assay coefficient of variation (CV) was 2.98% (range: 1.15–5.19%) for Pfim3 poly-14C detection in the different isolates, indicating good reproducibility in quantification. As our PCR/LDR assay enabled multiplex LDR, it could be useful for analyzing bacterial phase variation and performing epidemiological investigations.

*B. pertussis* fimbriae are phase-variable by altering poly(C) length in the promoter region. In this manner, phenotypic diversity could be created in the absence of genomic plasticity^23^. Nevertheless, there was long-term predominance of *B. pertussis* strains producing high Fim3 levels (Fig. 5). There may be selective advantages in Fim3 production for the *B. pertussis* strains circulating in Japan. This characteristic may enable *B. pertussis* strains to evade vaccine-driven selective pressure. Serotype shifts in *B. pertussis* have been reported for the UK, Finland, and France in response to pertussis vaccines containing fimbrial components^6,24,25^. Japan has been immunizing with the acellular pertussis vaccine (ACV) since 1981. Four ACV brands produced from *B. pertussis* Tohama I were available during the study period and two of them contained Fim2 as a minor antigen^26^. Anti-Fim2 immunity increased in the Japanese population following extensive ACV administration. This process may have driven Fim3 strain selection. However, there might be a trade-off response between *B. pertussis* virulence and fitness. The Fim2 and Fim3 strains differ in terms of virulence^27,28^. An epidemiological study in the UK demonstrated that Fim2 strain can cause a more severe disease than Fim3 strain^29^. High virulence tends to increase transmission but shortens the duration of infection which eventually reduces fitness^30^. The moderate virulence and high fitness of Fim3 might explain its persistent prevalence.

The present study examined Fim3-dependent autoagglutination in *B. pertussis* and established that it is strongly associated with the abundance of the Pfim3 poly-14C sequence and the amount of Fim3 production. Future studies should endeavor to elucidate the molecular mechanisms and clinical significance of the emergence of the *B. pertussis* Agg^+^ strain producing high levels of Fim3.

## Methods

### Bacterial strains, plasmids and growth conditions

The bacterial strains, plasmids, and PCR primers used in the present study were listed in Table S1. *B. pertussis* clinical isolates were randomly selected from our strain collection between 1994 and 2018 in Japan (n = 203), excepting strains have epidemiological links. *B. pertussis* strains were routinely grown on Bordet-Gengou (BG) agar plate or cyclodextrin solid medium (CSM) at 36 °C for 4 days^31^. Ampicillin (100 µg/mL), cephalexin (20 µg/mL), kanamycin (25 µg/mL), streptomycin (30 µg/mL), tetracycline (12.5 µg/mL) and/or sucrose (7.5% (w/v)) were added to the media as required for mutant selection purpose.

### Autoagglutination assay

*B. pertussis* strains were cultured on CSM with appropriate antibacterial agents, and then suspended in 1%(w/v) casamino acid solution in 0.6% NaCl (pH 7.1) to OD_650_ = 1.0. The bacterial suspensions were statically incubated in disposable cuvettes at 36 °C. Because a longer incubation does not contribute to a further classification of autoagglutination phenotype, we selected a 5-h incubation in this assay. The OD_650_ for each suspension was measured every 20 min for the first 2 h and then every 60 min up until 5 h.

### WGS

The whole bacterial genome was sequenced on a PacBio RSII sequencer (Pacific Biosciences, Menlo Park, CA, USA). Eight cells were used for single-molecule, real-time (SMRT) DNA sequencing, which was performed by the Dragon Genomics Center (Takara Bio Inc., Shiga, Japan). The sequences were assembled with SMRT Analysis v. 2.3 (Pacific Biosciences). Illumina MiSeq sequencing was also performed on *B. pertussis* BP300 and BP300s at DNA Chip Research Inc., Tokyo, Japan. The 150-bp short reads were used to validate the WGS in CLC Genomics Workbench v. 8.5.1 (Qiagen, Hilden, Germany). Genomes were compared with MUMmer v. 3.2337 (https://github.com/mummer4/mummer)^32^ and Mauve v. 2.4.038 (https://darlinglab.org/mauve/mauve.html)^33^.

### Immunoblot analysis

Total protein was extracted from the bacterial cells with sodium dodecyl sulfate (SDS)-lysis buffer. The protein samples were subjected to SDS–polyacrylamide gel electrophoresis (PAGE), transferred to nitrocellulose membranes (Bio-Rad Laboratories, Hercules, CA, USA), and incubated with rabbit anti-Fim2 and anti-Fim3 IgG polyclonal antibodies (Cusabio Biotech, Wuhan, China). Antigen–antibody complexes were visualized with horseradish peroxidase (HRP)-conjugated secondary antibody (Bio-Rad Laboratories) and Western Lightning ECL Pro reagents (PerkinElmer, Waltham, MA, USA). The blots were imaged with a LAS-3000 (Fujifilm, Tokyo, Japan). The original blots in this study are shown in Supplementary Fig. S4.

### Construction of B. pertussis ∆fim3 mutant

The fim3-deficient mutant was constructed by double-crossover homologous recombination as previously described, with minor modifications^34^. The Δfim3 sequence containing a 393-bp deletion was constructed by overlap extension PCR using *B. pertussis* BP300 genomic DNA as the template. A 1.3-kbp and a 1.2-kbp DNA fragment were amplified by PCR using attB1-fim3 and fim3-MP1 as well as fim3-MP2 and attB2-fim3R primers, respectively. The DNA fragments were joined by overlap extension PCR using the attB1-fim3F and attB2-fim3R primers. A third PCR was performed using the attB1-adaptor and attB2-adaptor primers with the second PCR product as the template. The final PCR product was cloned into pDONR221 by the adaptor PCR method and the Gateway cloning system (Invitrogen, Waltham, MA, USA) to obtain pDONR221-Δfim3. The pDONR221-Δfim3 and pABB-CRS2 vectors were combined in the Gateway cloning system to obtain pABB-Δfim3 which was then introduced into *E. coli* SM10λ*pir* and transconjugated into streptomycin-resistant *B. pertussis* BP300 (BP300Sm^r^). The mutant product was designated BP300Sm^r^Δfim3. Immunoblot analysis was used to confirm the lack of Fim3 protein production in the strain.

### Serotyping

Fim2 and Fim3 production were determined using enzyme-linked immunosorbent assay (ELISA) using anti-Fim2 and anti-Fim3 monoclonal antibodies (NIBSC, UK) as previously described^8^. The monoclonal antibodies were labelled with biotin to enable detection in a biotin-streptavidin system (Immuno-Biological Laboratories Co., Ltd., Gunma, Japan).

### PCR/LDR assay

The distribution of poly(C) in the *fim3* promoter was analyzed using PCR/LDR plus GeneScan^23^. The oligonucleotides used in this assay are listed in Table S1. PCR amplification was performed with Phusion High-Fidelity DNA polymerase with HF buffer according to the manufacturer’s instructions (New England Biolabs (NEB), Ipswich, MA, USA). The Pfim3-F and Pfim3-R primers were used to amplify the Pfim3 poly(C) region. The PCR conditions were as follows: 30 s at 98 °C followed by 30 cycles of 98 °C for 5 s, 55 °C for 10 s, 72 °C for 5 s, and final extension at 72 °C for 5 min. Each LDR reaction consisted of 1 μL of a PCR reaction, 8 U Taq DNA Ligase (NEB), 500 fmol labeled common oligonucleotide, and 500 fmol of a single unlabeled discriminating oligonucleotide in Taq DNA ligase reaction buffer (NEB) for a total volume of 20 µl. LDR oligonucleotides were synthesized and purified using HPLC (Sigma-Aldrich Corp., St. Louis, MO, USA). The reaction mixtures were heated at 95 °C for 2 min followed by 30 cycles of 95 °C for 15 s and 58 °C for 2 min. The reactions were stopped with 1.5 µL of 0.5 mM ethylenediaminetetraacetic acid (EDTA). Then 0.5 µL synthesized fragments was mixed with 0.2 µL GeneScan 120 LIZ size standard (Applied Biosystems, Foster City, CA, USA) plus 9.3 µL of Hi-Di formamide and heated to 95 °C for 3 min. The samples were immediately cooled on ice and analyzed either with a 3130xl genetic analyzer (Applied Biosystems) or a 3730 DNA analyzer (Applied Biosystems). The data were analyzed with GeneMapper software v. 4.0 (Applied Biosystems), and the abundance ratio of each poly(C) length was calculated from the peak area.

*B. pertussis* Tohama I genomic DNA with a major length of Pfim3 poly-13C was used as a control template to validate the accuracy of the fragment lengths determined by capillary electrophoresis. The GeneScan analysis returned fragment sizes that were smaller than those of the actual fragments, and calibration was required to map each peak to a corresponding poly(C) length. Repeated analyses of the lengths of poly-11C to poly-16C disclosed a mean coefficient of variation (CV) of 0.29% (range: 0.16–0.61%), and S.D. within ± 0.3 nucleotides.

### Statistical analysis

GraphPad Prism v. 9 (GraphPad Software, La Jolla, CA, USA) was used for all statistical analyses. *P* < 0.05 was considered statistically significant.

### Ethics statement

This article does not contain any studies involving human subjects performed by any of the authors.

## Supplementary Information

Supplementary Information 1.

Supplementary Information 2.

## Data Availability

The nucleotide sequence analyzed in this study were deposited at DDBJ Sequenced Read Archive (DRA) under the accession No. DRA009656 (https://ddbj.nig.ac.jp/resource/sra-submission/DRA009656).
