# Supplementary material for: Fim3-dependent autoagglutination of Bordetella pertussis
Source: Sci Rep. 2023 May 10;13:7629. doi: 10.1038/s41598-023-34672-0 (PMC10172299; doi:10.1038/s41598-023-34672-0)
Supplement: Supplementary file 1 — Supplementary Information 1. [file 41598_2023_34672_MOESM1_ESM.pdf]

Fim3-dependent autoagglutination of *Bordetella pertussis*

Nao Otsuka[1,*], Kentaro Koide[1], Masataka Goto[1], Kazunari Kamachi[1], Tsuyoshi Kenri[1]

[1]Department of Bacteriology II, National Institute of Infectious Diseases, Tokyo, Japan

*Corresponding author:

Nao Otsuka, PhD

Department of Bacteriology II

National Institute of Infectious Diseases, Japan

4-7-1 Gakuen

Musashimurayama, Tokyo 208-0011, Japan

Tel: +81-42-848-7101

Fax: +81-42-561-7173

E-mail: notsuka@niid.go.jp

Table S1. Bacterial strains and plasmids for genetic engineering

	Name	Characteristics	Reference
Strain	*B. pertussis* Tohama I	Japanese clinical isolate in 1952, and widely used as laboratory strain. Agg^- phenotype, MT87 carrying *ptxP1, ptxA2, prn1*, and *fim3A* alleles, $Fim2^+$/$Fim3^-$	Kamachi, K. et al. *Biologicals* 2009 Vol. 38 Pages 290-293
	B. pertussis BP300	Japanese clinical isolate in 2005, strong Agg^+ phenotype, MT26 carrying *ptxP3, ptxA1, prn2* and *fim3B* alleles, $Fim2^-$/$Fim3^+$	This study
	B. pertussis BP300s	Agg- phenotype, derivative of BP300	This study
	B. pertussis BP300Sm^r	Spontaneous streptomycin-resistant derivative of BP300, Sm^r	This study
	B. pertussis BP300$Sm^r\Delta$fim3	fim3 disrupted derivative of BP300Sm^r, Sm^r	This study
	E. coli DH5α	Host strain used for general genetic methods	Nippon gene
	E. coli SM10λpir	thi thr leu tonA lacY supE recA::RP4-2-Tc::Mu Km λpir	Simon R et al., *Nat Biotech*. 1983 Vol. 1 Issue 9 Pages 784-791
Plasmid	pDONR221	Cloning vector for Gateway system, Km^r, *ccdB*	Invitrogen
	pABB-CRS2	Low copy cloning vector for conjugation, Amp^r, *sacB*	Sekiya K et al., *Proc Natl Acad Sci* 2001 Vol. 98 Issue 20 Pages 11638-43

Table S2. Primers, probes, and oligonucleotides used in this study

Application	Name	Sequence (5' to 3')	Reference
DNA sequencing	M13 Primer M3	GTAAAACGACGGCCAG	universal primer
	M13 Primer RV	CAGGAAACAGCTATGAC	universal primer
	1567BF	GAAGGCGGCGACTGCATGA	This study
	1567CF	CTCGTGCTGGATAGGCGCAG	This study
	1567CF'	CTGCGCCTATCCAGCACGAG	This study
	1567R'	CGGCCTGCAACAAGAACGAA	This study
	1567R	TTCGTTCTTGTTGCAGGCCG	This study
	fim3-F1	ATGTCCAAGTTTTCATACCC	This study
	fim3-innerR	GGCTTGCGTGGTTTTGTC	This study
	fim3-innerR'	GACAAAACCACGCAAGCC	This study
	fim3-R	TCACGGTGCTCGACGCCAA	This study
	fim3-R'	TTGGCGTCGAGCACCGTGA	This study
	1569AF	GTCTTCCTTGCCGTGGGTC	This study
	1569AF'	GACCCACGGCAAGGAAGAC	This study
Δfim3 mutant construction	attB1-fim3F	AAAAAGCAGGCTGGCGACTGCATGACGATCTC	This study
	MP1-fim3	GCTTGCGTGGTTTTGTCCGTCTACTAGGGCGAGGCGGCAAGGATAA	This study
	MP2-fim3	TTATCCTTGCCGCCTCGCCCTAGTAGACGGACAAAACCACGCAAGC	This study
	attB2-fim3R	AGAAAGCTGGGTTCCGGAACCGCGACCTACT	This study
	attB1-adaptor	GGGGACAAGTTTGTACAAAAAAGCAGGCT	This study
	attB2-adaptor	GGGGACCACTTTGTACAAGAAAGCTGGGT	This study
PCR/LDR for Pfim3 poly(C)	Pfim3-F	AGGCCATTTTCATTGCGCGA	This study
	Pfim3-R	CTGATACTGAGGGGTGCCGT	This study
	fim3-11C	ACAACCATCAGCCC	This study
	fim3-12C	AAACAACCATCAGCCCC	This study
	fim3-13C	AAAAACAACCATCAGCCCCC	This study
	fim3-14C	AAAAAAACAACCATCAGCCCCCC	This study
	fim3-15C	AAAAAAAAACAACCATCAGCCCCCCC	This study
	fim3-16C	AAAAAAAAAAACAACCATCAGCCCCCCCC	This study
	fim3-common	Phos-CCCCCCCCGGACCTGATATTCTGATG-FAM	This study

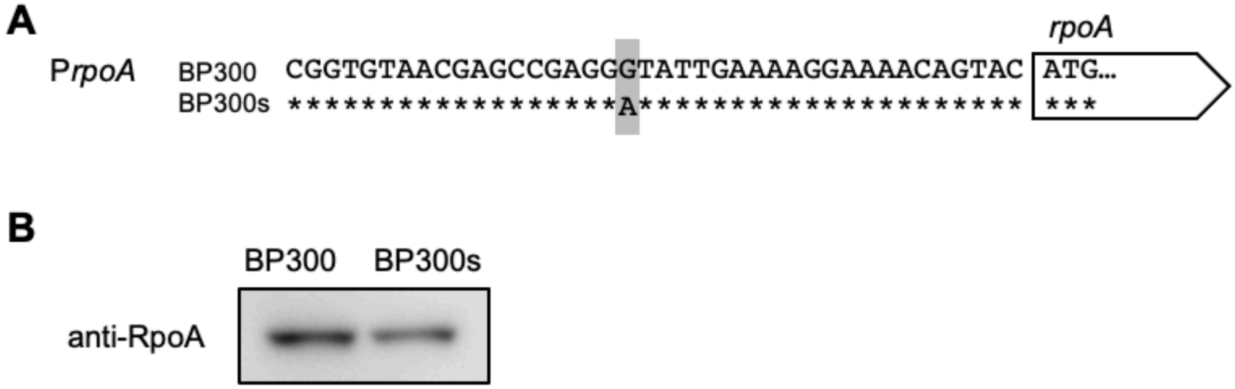

Fig S1. Single-point mutation in promoter region of *rpoA* gene found in Agg^{-} mutant, BP300s.

(A) Upstream sequence of *rpoA* gene is shown, and the SNP position is gray-highlighted. (B) Immunoblot analysis with anti-RpoA antibody. Mouse anti-*E. coli* RNA polymerase α (RpoA) was obtained from BioLegend (CA, USA).

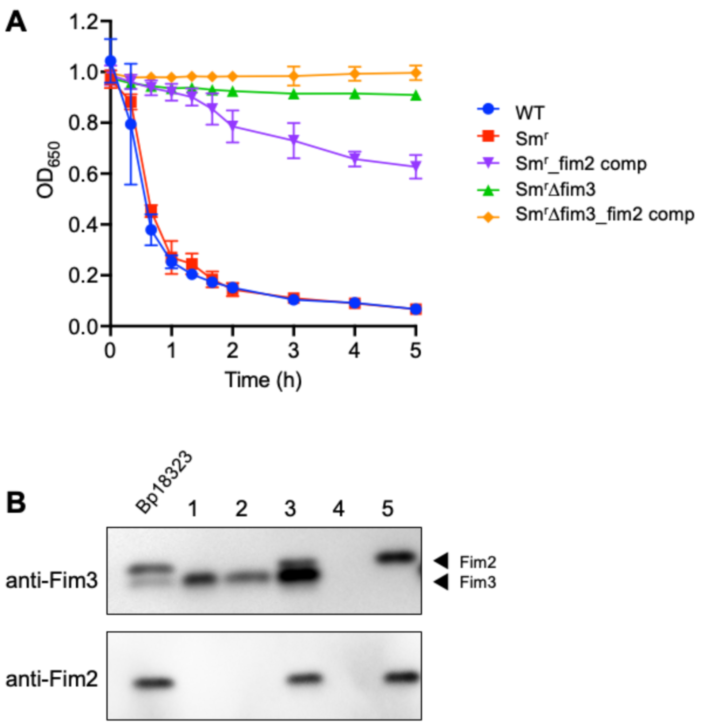

Fig. S2. The role of Fim2 in *B. pertussis* autoagglutination. (A) Autoagglutination assay of *B. pertussis* Δfim3 mutant and *fim2* gene-complemented mutants. (B) Immunoblot analysis using anti-Fim2 and anti-Fim3 antibodies. Rabbit anti-Fim2 immunoglobulin G (IgG) (number CSB-PA356626LA01BUA) and anti-Fim3 IgG (number CSB-PA322971LA01BUA) antibodies were purchased from Cusabio, China. *B. pertussis* BP300 (lane 1), BP300Sm^r (lane 2), BP300Sm^r/pRK-fim2 (lane 3), BP300Sm^{r}Δfim3 (lane 4), and BP300Sm^{r}Δfim3/pRK-fim2 (lane 5). Anti-Fim3 IgG reacts with both Fim2 and Fim3. Upper and lower arrows indicate Fim2 (22.5 kDa) and Fim3 (22.0 kDa), respectively. *B. pertussis* 18323 produces both Fim2 and Fim3 and is shown as a molecular standard.

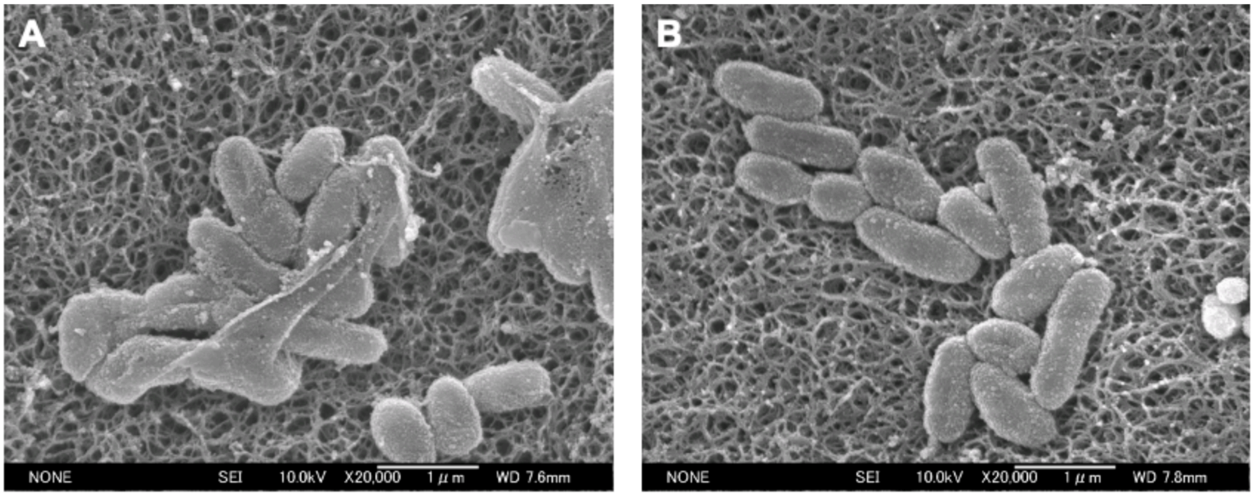

Fig. S3. SEM images of *B. pertussis* Agg^{+} strain and Agg^{-} mutant.

(A) *B. pertussis* Agg^{+} strain, BP300, (B) *B. pertussis* Agg^{-} mutant, BP300s. For analysis of cell morphology, 10^5 cells/10 µL of *B. pertussis* strains were spotted on Bordet–Gengou (BG) agar on the glass coverslips and incubated at 36 ºC overnight. Scanning electron microscopy (SEM) was performed on a JEOL JSM-6700F microscope (Tokyo, Japan).

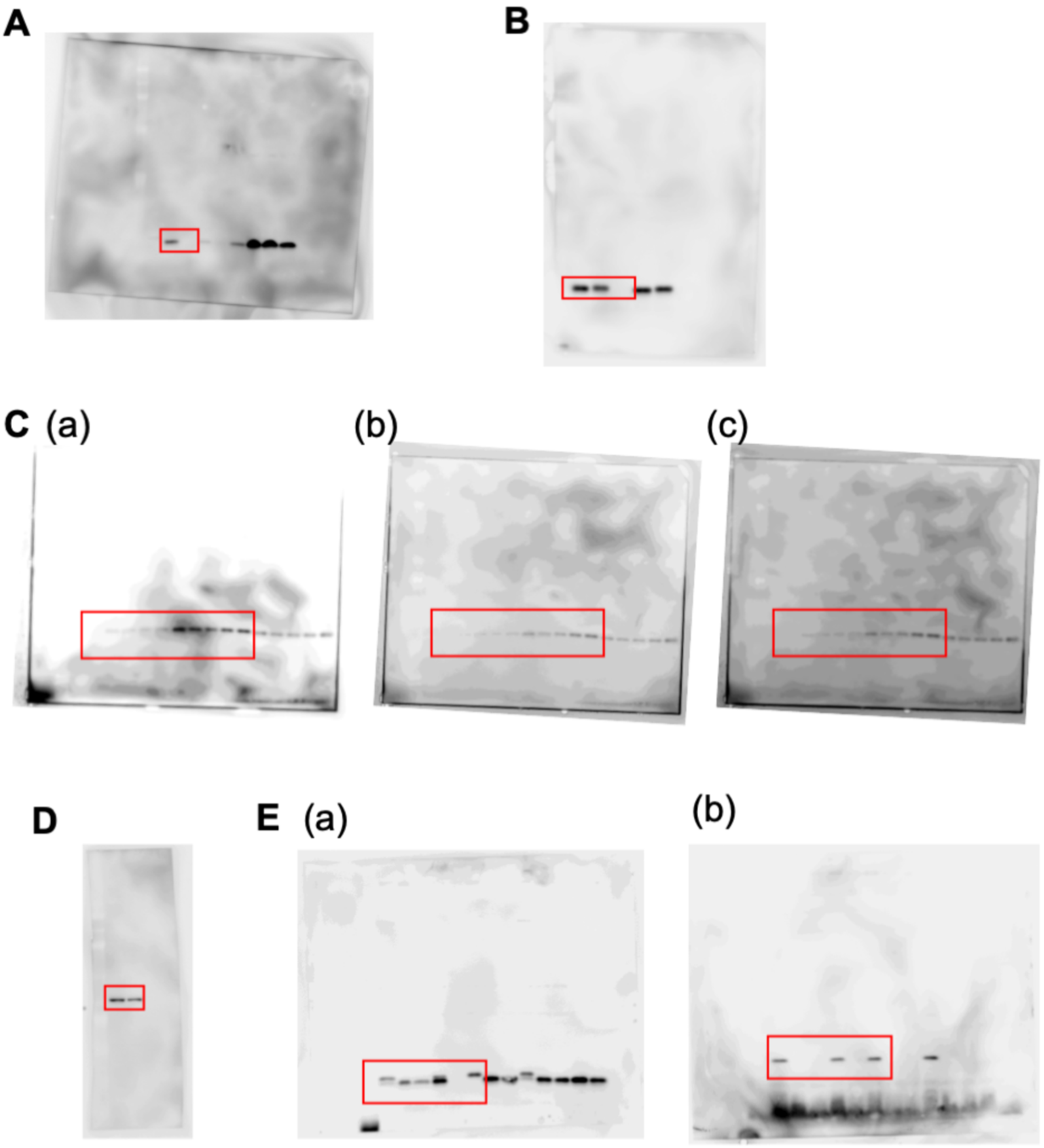

Fig. S4. The original blots in this study. (A) The original full-length blot of Figure 2D, (B) The original full-length blot of Figure 3A, (C) The blots shown in Figure 4; (a) the full-length blot in Figure 4, (b) and (C) are blots with different exposure or contrast, (D) The original full-length blot of Figure S1B, (E) The original full-length blots of Figure S4B; (a)

anti-Fim3 detection, (b) anti-Fim2 detection. The red-lined areas are clopped and shown in each figure.
